# Predictors of Intention to Stay in Healthcare Among Healthcare Students and Professionals in Czechia and Slovakia

**DOI:** 10.1155/jonm/1300076

**Published:** 2026-08-07

**Authors:** Michal Vostrý, Vlastimil Chytrý, Aneta Hujová, Lucia Mičíková, Kateřina Kovářová, Barbora Lanková, Katarína Kašlíková

**Affiliations:** ^1^ Faculty of Health Studies, Jan Evangelista Purkyně University in Ústí nad Labem, Ústí nad Labem, Czech Republic, ujep.cz; ^2^ Faculty of Education, Jan Evangelista Purkyně University in Ústí nad Labem, Ústí nad Labem, Czech Republic, ujep.cz; ^3^ Faculty of Healthcare, Alexander Dubček University of Trenčín in Trenčín, Trenčín, Slovakia

**Keywords:** cross-national comparison, Czech Republic, healthcare students, healthcare workforce, intention to stay, leadership, professional commitment, Slovakia, workplace relationships

## Abstract

**Background:**

Workforce stability remains a major challenge for sustainable healthcare systems. Retention research increasingly distinguishes between organizational conditions, professional identification, and mobility or place‐related preferences. However, less is known about how these factors operate within closely related Central European contexts and in samples that include respondents connected to healthcare education and practice. This study examined whether workplace relationships and leadership, professional commitment, and place‐related mobility preferences predict intention to stay in healthcare and compared these associations in the Czech Republic and Slovakia.

**Methods:**

A descriptive, correlational, cross‐sectional study was conducted among healthcare students and professionals in the Czech Republic and Slovakia. The analytical sample included 2103 respondents, comprising 1011 participants from the Czech Republic and 1092 from Slovakia. Data were collected using an author‐developed questionnaire focused on workplace relationships and leadership, professional commitment, place‐related mobility preferences, and related aspects of the work and educational environment. Exploratory and confirmatory factor analyses were used to examine the latent structure of the instrument and assess reliability, followed by multiple linear regression and cross‐national comparison.

**Results:**

The final model supported three interpretable dimensions: workplace relationships and leadership, professional commitment, and place‐related mobility preferences. In the Czech and Slovak samples, the regression models explained 23.2% and 23.3% of the variance in intention to stay, respectively. In the regression analyses, Q41 was used exclusively as the dependent variable and was not included in the professional‐commitment predictor composite. Within the tested model, professional commitment showed the largest standardized association with intention to stay in both national samples, workplace relationships and leadership showed a significant but smaller association, and place‐related mobility preferences were not statistically significant predictors. Although the omnibus country comparison reached statistical significance, differences in individual dimensions were trivial or very small, indicating substantial similarity between the Czech and Slovak samples.

**Conclusion:**

In these cross‐sectional data, intention to stay in healthcare was more strongly associated with professional commitment than with workplace relationships and leadership or place‐related mobility preferences. The findings should be interpreted with caution because the outcome was assessed using a single, conditionally worded item and because the sample included respondents connected to both healthcare education and practice. Nevertheless, the results suggest that retention strategies may benefit from combining improvements in organizational conditions with deliberate support for professional meaning, commitment, and identification with the healthcare role.

**Implications for Nursing Management:**

The findings suggest that nursing managers, educators, and healthcare leaders should not rely solely on structural or logistical retention measures, such as staffing arrangements, geographic flexibility, or financial incentives. Although supportive workplace relationships and leadership remain important, the largest association with intention to stay was observed for professional commitment within the tested model. Retention strategies should therefore include systematic efforts to strengthen professional meaning, value congruence, identification with the healthcare role, mentoring, recognition, and supportive leadership practices. Nursing management interventions may be most effective when they create working and learning environments that reinforce professional commitment rather than addressing workplace conditions in isolation.

## 1. Introduction

Workforce stability has become one of the central prerequisites for sustainable health systems. In nursing and other healthcare professions, this issue is not limited to the absolute number of available workers but also concerns whether current and future members of the workforce are willing and able to remain in the field over time. Workforce stability is therefore understood here as the capacity of healthcare systems and organizations to sustain a sufficiently prepared, committed, and continuing workforce. Contemporary reviews show that retention is a multidimensional phenomenon shaped by organizational conditions, professional experience, personal resources, and broader labor‐market factors, with consequences for staffing continuity, team functioning, and quality of care [1–6]. Intention to stay is a useful early indicator of this process because it captures respondents’ stated willingness to continue in healthcare before actual turnover or career exit occurs [5, 7]. Existing research links this intention to interpersonal safety at work, communication openness, staffing conditions, workload, leadership quality, and the wider practice environment [8–13]. At the same time, the effects of the work environment on workforce stability may not be only direct. Burnout, subjective well‐being, professional values, and perceived meaning in work can mediate or modify how organizational conditions are translated into decisions about staying or leaving [14–16]. For this reason, retention needs to be examined as a combination of relational, professional, and contextual factors rather than as a single organizational problem.

The present study focuses on three domains that are both theoretically relevant and practically important for retention. Workplace relationships and leadership refer to the quality of interpersonal climate, communication, trust, feedback, supervisor support, and shared responsibility in everyday work or clinical‐learning settings. Professional commitment refers to the degree to which respondents perceive healthcare work as meaningful, value‐congruent, developmental, and worth continuing. Mobility is treated more narrowly as a place‐related preference dimension, reflecting orientation toward work abroad, preference for working close to home, and willingness to relocate for a better offer. Previous studies suggest that professional identity, career calling, resilience, transition experience, and professional values are closely connected with retention intention, especially among students, trainees, and early‐career nurses [17–21]. Mobility and migration intentions are also relevant, particularly in Central and Eastern European contexts, but the literature suggests that such intentions are intertwined with professional identity, financial incentives, career opportunities, and learning environments rather than operating as simple standalone predictors [22–24].

A further rationale for the present study is the comparison between the Czech Republic and Slovakia. These countries share historical, linguistic, educational, and professional proximity, while functioning as independent healthcare systems with their own institutional arrangements. This creates a useful comparative setting: Substantial similarity between samples would suggest that the studied mechanisms may be robust across closely related contexts, whereas meaningful differences would indicate that national or institutional conditions shape retention‐related attitudes. The comparison was therefore treated as an empirical question rather than as an assumption of strong divergence. By examining workplace relationships and leadership, professional commitment, and place‐related mobility preferences in one model, the study aims to provide a clearer basis for workforce‐stabilization strategies directed at both current and future healthcare personnel.

### 1.1. Aim of the Study

The aim of this study was to analyze factors associated with intention to remain in healthcare among respondents connected to healthcare education and practice in the Czech Republic and Slovakia, with particular emphasis on workplace relationships and leadership, professional commitment, and place‐related mobility preferences. The study focused on identifying and validating the factor structure of these dimensions through exploratory and confirmatory factor analysis (CFA), assessing their reliability, and quantifying their associations with intention to remain in healthcare by means of regression modeling. A further objective was to compare the identified relationships between the Czech and Slovak samples and to examine whether the pattern of associations was similar across two closely related national contexts. The study thus seeks to provide empirically grounded insights for human‐resource, educational, and organizational strategies aimed at supporting workforce stability in healthcare.

### 1.2. Research Hypothesis

• H1: Professional commitment, workplace relationships and leadership, and place‐related mobility preferences are statistically significantly associated with respondents’ intention to remain in healthcare.
• H2: The Czech and Slovak samples differ in the observed dimensions and/or in the relationships between these dimensions and the intention to remain in healthcare. Given the close historical and institutional proximity of the two countries, this hypothesis was tested as an exploratory comparative question rather than as an assumption of large between‐country differences.

## 2. Methods

This study was conducted as a descriptive, correlational, and cross‐sectional study. The chosen design made it possible both to describe the levels of the variables under investigation and to analyze relationships among them. The research focused on selected dimensions of the work and educational environment, professional orientation, and intention to remain in healthcare. Data were collected through a questionnaire survey that included items on workplace relationships and leadership, professional commitment, place‐related mobility preferences, and related aspects of healthcare work and preparation for healthcare careers. The questionnaire used in this study is provided in the supporting information. Subsequently, the data were subjected to multivariate statistical analyses, including exploratory and CFA and multiple linear regression analysis. The Czech Republic and Slovakia were selected because they represent two closely related yet independent national contexts. Their proximity allows for a meaningful comparison without the strong distortion that may arise when culturally or institutionally distant systems are compared, while their separateness makes it possible to examine whether the observed relationships are stable across national contexts. The questionnaire is a supporting 1 this article.

### 2.1. Population and Sample

The target population comprised respondents connected to healthcare education or practice in the Czech Republic and Slovakia, including healthcare students or trainees as well as current healthcare workers and professionals. For this reason, the revised manuscript uses the broader term healthcare students and professionals rather than treating the sample as a homogeneous group of fully employed healthcare professionals. Participants were recruited by nonprobability convenience sampling through collaborating healthcare‐related educational and professional institutions and institutional contacts. The questionnaire was self‐administered. Because the invitation was distributed through institutional channels and the exact number of individuals who received it could not be determined reliably, an exact response rate could not be calculated. Eligible respondents were persons connected to a healthcare study or healthcare work who provided informed consent and completed the key questionnaire items required for the analyses. The variable originally labeled gender in the questionnaire is reported in the manuscript as self‐reported sex/gender category, because the retained analytical categories were male and female. The Czech sample comprised 1011 respondents (men: *n* = 127; 12.5%, women: *n* = 884; 87.5%). The Slovak sample comprised 1092 respondents (men: *n* = 184; 16.8%, women: *n* = 908; 83.2%). Only respondents with complete data for the key variables were included in the analysis, which corresponds to the listwise deletion procedure used in the multivariate statistical methods. This approach ensured the consistency of composite scores and subsequent analyses. In both samples, the sex/gender structure was markedly asymmetric in favor of women, which corresponds to the typical distribution in many healthcare and nursing‐related educational and professional settings. Missingness was largely block‐wise rather than item‐specific. In the Czech dataset, 276 records had missing values on all key questionnaire items and did not contain usable demographic information. In the Slovak dataset, 163 records did not meet the sex/gender‐valid sample definition; among the retained Slovak sample, two records were incomplete for the final regression model. Because the missing data primarily reflected incomplete questionnaire blocks and because the main analyses required consistently defined composite scores, listwise deletion was used. Multiple imputation was not used as the primary approach because imputing nonstarted or substantially incomplete questionnaire blocks would require stronger assumptions and could introduce artificial information into scale construction. Because most excluded Czech records contained no usable questionnaire or demographic information, formal comparison between complete and incomplete cases was not meaningful. In Slovakia, exclusions mainly reflected the predefined sex/gender‐valid analytic sample rather than item‐level missingness. The complete‐case approach therefore ensured that all psychometric and regression analyses were based on consistently defined observed item responses. At the same time, the size of both samples met and substantially exceeded the recommended minimum requirements for the application of factor analysis and regression modeling, thus allowing for sufficient statistical power and robust inferential conclusions. The sample size was assessed with regard to the requirements for precise estimation of population parameters. Assuming a population size of approximately 25,000 individuals, a 95% confidence level, and a maximum permissible error of ±5%, the minimum recommended sample size is *n* = 379. However, the research sample included more than *n* > 1000 respondents in each national sample, which exceeds this threshold by more than double. From the perspective of inferential statistics, lower standard errors of estimation and greater precision of model parameters can therefore be expected. The sample size also meets common recommendations for multivariate methods, especially factor analysis and structural modeling, where at least 5–10 observations per variable are recommended [25] or, alternatively, an absolute sample size above 200 is considered sufficient for stable estimates [26]. In the context of CFA, a larger sample size additionally contributes to the robustness of parameter estimates and the stability of the covariance structure, although it simultaneously increases the sensitivity of the chi‐square test, which may lead to statistical significance even when the model demonstrates relatively good fit to the data. Overall, the sample size provides high statistical power and allows for reliable inferential conclusions.

### 2.2. Data Analysis

The questionnaire was developed by the authors for the purposes of this study. The decision to use an author‐developed instrument was based on the aim of capturing a context‐specific combination of retention‐related preferences relevant to both healthcare education and healthcare practice in two closely related Central European settings. Existing validated instruments often focus on narrower constructs, such as turnover intention, organizational commitment, practice environment, or job satisfaction, whereas the present study required a brief instrument covering workplace relationships and leadership, professional commitment, and place‐related mobility preferences within one questionnaire. Item content was informed by the retention and nursing‐workforce literature and by the authors’ expertise in healthcare education, healthcare management, and applied educational research. Items were formulated as direct statements and answered on a seven‐point agreement scale. The central outcome, Q41, was intentionally treated as a single, conditionally worded indicator of intention to stay; this measurement decision is explicitly acknowledged as a limitation in the Discussion. Data quality was supported by range checks, exclusion of records with missing key questionnaire blocks, and listwise use of complete cases for the analyses. No separate attention‐check items were embedded in the questionnaire, which is also acknowledged as a limitation. Statistical analyses were conducted using IBM SPSS Statistics and JASP software. Prior to the main analyses, the suitability of the data for the application of multivariate statistical methods was verified. Normality of distribution was assessed using the Shapiro–Wilk test, and homogeneity of variances was examined using Levene’s test. Given the size of the samples, the interpretation of normality tests was supplemented by an assessment of distributional shape as well as skewness and kurtosis values. The data structure was further analyzed using exploratory factor analysis (EFA), performed by means of principal axis factoring with oblimin rotation. The number of factors was determined based on a combination of Kaiser’s criterion, parallel analysis, and substantive interpretability. Subsequently, the factor structure was verified using CFA with the DWLS estimator, which is appropriate for ordinal data. The internal consistency of the identified scales was assessed using Cronbach’s alpha (*α*) and McDonald’s omega (*ω*), while the split‐half method with Spearman–Brown correction was used as a complementary approach. These indicators were interpreted with regard to the length of the scales and the exploratory nature of the research. Correlation analyses were used to test the relationships among variables. Depending on whether the assumptions were met, Pearson correlation coefficients (*r*) were calculated for approximately normally distributed variables, while Spearman’s rank‐order correlations (*ρ*) were used for data that did not meet the assumptions of normality. To assess differences between the Czech and Slovak samples, a multivariate approach was first applied using Hotelling’s *T* ^2^ test. Subsequently, independent‐samples *t*‐tests were conducted for the individual variables. In cases where the assumptions of normality were violated, nonparametric alternatives were used, specifically the Mann–Whitney *U* test. When multiple testing was performed, a correction of the significance level was taken into account, for example, the Bonferroni correction. To identify the determinants of intention to remain in healthcare, multiple linear regression analysis was applied separately in the Czech and Slovak samples. Scale scores were calculated as sum scores. Workplace relationships and leadership were calculated from Q20, Q21, Q22, Q23, Q29, and Q30. Professional commitment was calculated from Q5, Q8, Q11, and Q12 for the regression analyses. Although Q41 loaded on the professional‐commitment factor in the measurement analyses, it was not included in the professional‐commitment predictor score because Q41 served as the dependent variable. This prevented criterion contamination. Mobility was calculated from Q7, Q32, and Q33. The scale was interpreted as a place‐related mobility‐preference dimension, reflecting respondents’ orientation toward geographical aspects of future work, including working abroad, working close to home, and willingness to relocate for a better job. The results were interpreted on the basis of both unstandardized and standardized regression coefficients; however, the text focuses primarily on standardized coefficients, while full unstandardized results are presented in the regression table.

### 2.3. Assumptions of Factor Analysis

Prior to the main analysis, the suitability of the data for factor analysis was verified. The Kaiser–Meyer–Olkin index reached KMO = 0.793, which corresponds to good data quality. The MSA values of the individual items ranged from 0.627 to 0.889, with most items reaching values above 0.70, thus confirming their adequacy for factor analysis. Bartlett’s test of sphericity was statistically significant (*χ* ^2^ (153) = 4759; *p* < 0.001), indicating that the correlation matrix differs significantly from the identity matrix and contains sufficiently strong relationships among the items.

### 2.4. Exploratory Factor Analysis

EFA was conducted using principal axis factoring with oblimin rotation. Based on a combination of Kaiser’s criterion, parallel analysis, and substantive interpretability, a four‐factor model was identified. Subsequent item reduction based on factor loadings (≥ 0.40) led to the definition of four interpretable dimensions. The first factor included items related to interpersonal relationships and leadership (Q20, Q21, Q22, Q23, Q29, and Q30), which showed high factor loadings (0.456–0.649). This factor was interpreted as workplace relationships and leadership. The second factor consisted of items Q5, Q8, Q11, Q12, and Q41, which reflect intrinsic motivation, meaningfulness of work, and professional orientation. Factor loadings ranged from 0.407 to 0.616 in absolute values, while the negative sign merely reflects the direction of scaling. This factor was interpreted as professional commitment and meaningfulness of work. The third factor was clearly defined by items Q7, Q32, and Q33, which relate to respondents’ geographical mobility. Factor loadings ranged from 0.555 to 0.640, indicating high consistency of this dimension. The fourth factor included items Q25, Q35, Q39, and Q13, relating to financial remuneration and recognition. Factor loadings ranged from 0.407 to 0.555. Item Q13 was excluded from the final remuneration scale because it showed a lower factor loading, and its inclusion led to a reduction in the internal consistency of the scale. After its removal, reliability increased (*α* = 0.64), while the remaining items demonstrated greater content homogeneity. After applying the factor loading criterion of ≥ 0.40, four stable and substantively interpretable factors were identified, with Cronbach’s alpha values ranging from 0.62 to 0.73. The reliability of the individual scales is presented in the following Table 1.

**TABLE 1:** Internal consistency of individual scales.

Factor	Items included	Number of items	Cronbach’s alpha	McDonald’s omega	Analytical use
Workplace relationships and leadership	Q20, Q21, Q22, Q23, Q29, Q30	6	0.692	0.722	Measurement model and regression predictor
Professional commitment, measurement scale	Q5, Q8, Q11, Q12, Q41	5	0.706	0.707	CFA measurement model
Professional commitment, regression composite (Q41 excluded)	Q5, Q8, Q11, Q12	4	0.673	Not recalculated/not reported	Regression predictor
Place‐related mobility preferences	Q7, Q32, Q33	3	0.617	0.629	Measurement model and regression predictor
Compensation and recognition	Q25, Q35, Q39	3	0.641	0.652	Exploratory scale; not retained in final regression model

*Note:* Professional commitment in the measurement model included Q5, Q8, Q11, Q12, and Q41. For the regression analyses, Q41 was treated exclusively as the dependent variable and was therefore excluded from the professional‐commitment predictor composite. The regression composite for professional commitment was calculated from Q5, Q8, Q11, and Q12. Cronbach’s alpha for the four‐item professional‐commitment predictor was 0.673 overall, 0.671 in the Czech sample, and 0.676 in the Slovak sample. Place‐related mobility preferences were calculated from Q7, Q32, and Q33 and interpreted as a place‐related preference index rather than as a fully validated mobility scale.

The internal consistency of the individual scales was assessed using Cronbach’s alpha and McDonald’s omega. The observed values ranged from *α* = 0.617 to 0.706 and *ω* = 0.629 to 0.722, which may be considered acceptable in the context of exploratory social science research. Specifically, the scales workplace relationships and leadership (*ω* = 0.722; *α* = 0.692) and professional commitment (*ω* = 0.707; *α* = 0.706) reached values close to or exceeding the 0.70 threshold, which is traditionally regarded as the minimum level of acceptable internal consistency [27]. At the same time, these values are in line with recommendations according to which values around 0.70 may be considered sufficient, especially in exploratory research [25]. The mobility scale showed lower reliability values (*ω* = 0.629; *α* = 0.617), which should, however, be interpreted with regard to the small number of items. Cronbach’s alpha is sensitive to scale length, and in short instruments it may reach lower values without this necessarily indicating insufficient internal consistency; in such cases, other item characteristics, such as inter‐item correlations, should also be considered [28]. For newly developed or shorter scales, values in the range of 0.60–0.70 may be considered acceptable [25]. Similarly, the remuneration and recognition scale (*ω* = 0.652; *α* = 0.641) reached values slightly below the 0.70 threshold, yet still within the acceptable range for exploratory studies. Rigid application of universal cutoff values for Cronbach’s alpha is not methodologically appropriate, and the interpretation of reliability should be based on the broader research context and supported by additional evidence of measurement validity [29]. A supporting split‐half analysis produced consistent results, which further supports the stability of the measurement. Overall, the retained scales reached an acceptable level of reliability for exploratory use, while the higher values for workplace relationships and professional commitment strengthen confidence in the stability of the main findings. The lower values observed for the shorter scales require cautious interpretation but do not by themselves indicate a major measurement problem. It was found that the remuneration factor did not demonstrate sufficient stability across analyses. Although internal consistency increased after item reduction, CFA did not demonstrate adequate model fit to the data. For this reason, the factor was not included in the final model; however, its role was further examined exploratorily. CFA was conducted to verify the three‐factor structure without the remuneration factor. The model included the latent dimensions of workplace relationships and leadership (Q20, Q21, Q22, Q23, Q29, and Q30), professional commitment (Q5, Q8, Q11, Q12, and Q41), and mobility (Q7, Q32, and Q33). The analysis was carried out using the DWLS estimator, which is suitable for ordinal data. The test of model fit was statistically significant (*χ* ^2^(74) = 594.393; *p* < 0.001), which is expected given the sensitivity of the chi‐square test to sample size. The comparative fit index reached CFI = 0.888, and the Tucker–Lewis index reached TLI = 0.863. These values were below the conventional 0.90 threshold and therefore indicate borderline rather than optimal global fit. The RMSEA value of 0.083 (90% CI: 0.077–0.090) also suggested borderline approximation, whereas SRMR = 0.071 indicated acceptable local fit. All factor loadings were statistically significant (*p* < 0.001) and showed moderate to high values, supporting the substantive interpretability of the model. The interpretation of fit indices cannot be based on rigid universal cutoffs, because mechanical application of such thresholds may lead to erroneous conclusions [30]. Although some indices did not meet stricter recommendations, for example, CFI ≥ 0.95 [31], the values fall within a range often considered acceptable depending on research context, model complexity, and the nature of the data [32, 33]. The presented model was therefore interpreted as an empirically adequate but imperfect approximation of the construct structure. All latent factors showed statistically significant variances (*p* < 0.001), confirming their differentiation within the model. Correlations among the factors were generally low. A statistically significant relationship was found between workplace relationships and professional commitment (*r* = 0.147; *p* < 0.001), suggesting that a more positively perceived work environment is associated with a higher level of professional commitment. By contrast, the relationships between mobility and the other factors were not statistically significant. The residual variances of the individual items ranged across a relatively broad interval, suggesting that part of the variability in the items is not explained by the latent factors, which is common in psychological and social data. We considered possible model refinements, including correlated residuals within factors and alternative item sets, but did not introduce post hoc correlated residuals because they lacked a clear theoretical justification and could have led to overfitting. Similarly, additional item deletion was not applied because it would have weakened the substantive coverage of the constructs, particularly in the shorter scales. Standardized factor loadings are provided in Supporting Information 2, Table S1, to facilitate interpretation of the CFA measurement model.

### 2.5. Regression Analysis

To identify the determinants of intention to remain in healthcare, multiple linear regression analysis was conducted separately for the Czech and Slovak datasets. Q41 was used exclusively as the dependent variable. To avoid criterion contamination, Q41 was excluded from all predictor composites. The professional‐commitment predictor was therefore calculated from Q5, Q8, Q11, and Q12 only. Workplace relationships and leadership were calculated from Q20, Q21, Q22, Q23, Q29, and Q30. Mobility was calculated from Q7, Q32, and Q33. All models were estimated using listwise complete cases for the variables included in the respective regression model.

## 3. Results for the Czech and Slovak Datasets

Separate multiple linear regression analyses were conducted for the Czech and Slovak samples to examine whether workplace relationships and leadership, professional commitment, and place‐related mobility preferences predicted intention to stay in healthcare. The full unstandardized coefficients, standard errors, standardized coefficients, *p* values, and model fit statistics for both national models are presented in Table 2.

**TABLE 2:** Multiple linear regression predicting intention to stay in healthcare (Czech Republic and Slovakia).

Predictor	B CZ	SE CZ	*β* CZ	*p*	B SK	SE SK	*β* SK	*p*
Workplace relationships and leadership	0.059	0.008	0.210	< 0.001	0.056	0.007	0.208	< 0.001
Professional commitment (Q41 excluded)	0.123	0.009	0.381	< 0.001	0.123	0.009	0.385	< 0.001
Place‐related mobility preferences	−0.008	0.008	−0.026	0.349	0.003	0.008	0.010	0.709

*Note: B* = unstandardized coefficient; *β* = standardized coefficient. CZ = Czech Republic; SK = Slovakia. Czech model: *n* = 1011; *R* ^2^ = 0.232; adjusted *R* ^2^ = 0.230; *F* (3, 1007) = 101.28; *p* < 0.001. Slovak model: *n* = 1090; *R* ^2^ = 0.233; adjusted *R* ^2^ = 0.231; *F* (3, 1086) = 109.81; *p* < 0.001.

Abbreviation: SE = standard error.

The Czech regression model was statistically significant, *F* (3, 1007) = 101.28; *p* < 0.001, and explained 23.2% of the variance in intention to stay, *R* ^2^ = 0.232; adjusted *R* ^2^ = 0.230. In the Czech sample, professional commitment showed the largest standardized association with intention to stay, *β* = 0.381; *p* < 0.001, followed by workplace relationships and leadership, *β* = 0.210; *p* < 0.001. Place‐related mobility preferences did not emerge as a statistically significant predictor, *β* = −0.026; *p* = 0.349. The same substantive pattern was observed in the Slovak sample, where the model explained 23.3% of the variance in intention to stay, *R* ^2^ = 0.233; adjusted *R* ^2^ = 0.231. Professional commitment again showed the largest standardized association, *β* = 0.385; *p* < 0.001, followed by workplace relationships and leadership, *β* = 0.208; *p* < 0.001. Place‐related mobility preferences remained weak and statistically nonsignificant, *β* = 0.010; *p* = 0.709. Descriptive statistics for the main study variables are presented in Table 3.

**TABLE 3:** Descriptive statistics of study variables by country.

Variable	CZ *n*	CZ *M*	CZ SD	CZ median	CZ range	SK *n*	SK *M*	SK SD	SK median	SK range	*p*	Cohen’s d
Workplace relationships and leadership	1011	36.016	3.572	36	19–42	1092	36.248	3.738	37	18–42	0.146	−0.063
Professional commitment, measurement scale	1011	27.839	3.650	28	9–35	1090	27.873	3.663	28	9–35	0.828	−0.009
Professional commitment, regression composite, Q41 excluded	1011	21.783	3.100	22	4–28	1090	21.801	3.114	22	4–28	0.897	−0.006
Place‐related mobility preferences	1011	11.221	3.320	11	3–21	1092	11.558	3.468	12	3–21	0.023	−0.099
Compensation and recognition	1011	17.042	2.719	17	7–21	1092	16.835	2.821	17	6–21	0.088	0.074
Intention to stay, Q41	1011	6.055	1.003	6	1–7	1092	6.071	0.999	6	1–7	0.714	−0.016

*Note:* CZ = Czech Republic; SK = Slovakia; *M* = mean. *p* values are based on independent‐samples *t*‐tests. Cohen’s *d* expresses the standardized mean difference between the Czech and Slovak samples. Mobility was calculated from Q7, Q32, and Q33 and was interpreted as a place‐related mobility‐preference dimension. Q41 is reported separately as the dependent variable. The four‐item professional‐commitment regression composite excludes Q41 to avoid criterion contamination.

Abbreviation: SD = standard deviation.

Table 3 presents descriptive statistics for the main study variables by country. The Czech and Slovak samples showed highly similar mean values across the observed dimensions. Differences in workplace relationships and leadership, professional commitment, compensation and recognition, and intention to stay were negligible and statistically nonsignificant. Place‐related mobility preferences showed a nominally significant difference between the two samples; however, the corresponding effect size was very small, indicating limited practical relevance. These descriptive results support the interpretation that the Czech and Slovak samples were substantively comparable across the main study variables.

### 3.1. Differences Between the Countries in the Individual Dimensions Under Study

Differences between the Czech and Slovak samples were examined using Hotelling’s T^2^ test and follow‐up independent‐samples *t*‐tests. The omnibus test was statistically significant, indicating an overall multivariate difference between the two national samples. However, the univariate comparisons showed only trivial or very small differences. Workplace relationships and leadership did not differ significantly between countries, *p* = 0.146, *d* = −0.063. Professional commitment was virtually identical in the Czech and Slovak samples, *p* = 0.828, *d* = −0.009. Place‐related mobility preferences showed a nominally significant difference, *p* = 0.023, but the effect size was very small, *d* = −0.099, and the result did not remain robust after Bonferroni correction. Compensation and recognition also did not differ significantly, *p* = 0.088, *d* = 0.074. Overall, the country comparison suggests statistical sensitivity to minor differences in a large sample rather than substantively meaningful divergence between the Czech and Slovak respondents.

### 3.2. Refined Interpretation of the Findings

The psychometric analyses indicate that the final instrument captures three conceptually meaningful and empirically distinguishable domains relevant to intention to stay in healthcare: workplace relationships and leadership, professional commitment, and place‐related mobility preferences. The KMO value and Bartlett’s test supported the suitability of the data for factor analysis, and the retained scales showed reliability values acceptable for exploratory research. At the same time, CFA results indicated borderline rather than optimal model fit, and the mobility dimension showed weaker internal consistency because of its short three‐item structure. The measurement model should therefore be interpreted as a usable but imperfect approximation rather than as a fully established scale. The regression results provide evidence regarding the relative contribution of the studied predictors within the tested model. In both national samples, professional commitment showed the largest standardized association with intention to stay, workplace relationships and leadership showed a smaller but statistically significant association, and place‐related mobility preferences were not statistically significant. The models explained approximately 23% of the variance in intention to stay, which is meaningful for cross‐sectional workforce‐attitude research but also indicates that other determinants not included in the present model remain important. These findings justify cautious interpretation: Professional commitment appears central within this model, but it should not be treated as the sole or exhaustive explanation of the intention to stay.

## 4. Discussion

The present study contributes to the literature on healthcare workforce retention by examining workplace relationships and leadership, professional commitment, and place‐related mobility preferences within one comparative model. The findings suggest that, in both national samples, professional commitment had the largest standardized association with intention to stay, while workplace relationships and leadership had a significant but smaller association, and place‐related mobility preferences were not statistically significant. This pattern is consistent with research showing that retention is linked not only to staffing levels and organizational arrangements but also to job satisfaction, professional meaning, commitment, practice environment, recognition, and psychological resources [1, 2, 5, 7, 34]. Importantly, the explained variance of approximately 23% indicates that the model captured a meaningful but partial share of the retention‐related decision process. Therefore, the results should not be interpreted as showing that professional commitment fully determines intention to stay; rather, professional commitment appears to be the strongest predictor among the variables examined in this study.

The stronger association of professional commitment is theoretically plausible. Previous studies have linked professional identity, career calling, psychological capital, resilience, value orientation, and professional values with lower turnover intention or stronger retention intention, especially among students, trainees, and early‐career nurses [14, 17–21]. From this perspective, intention to stay is not only a reaction to immediate workplace conditions but also reflects whether respondents perceive healthcare as meaningful and compatible with their professional self‐concept. The significant but smaller role of workplace relationships and leadership is also consistent with current evidence. Supportive supervision, effective communication, psychologically safe climates, authentic or transformational leadership, and favorable practice environments have repeatedly been associated with better job attitudes and lower intention to leave [10–13, 35, 36]. These findings suggest that leadership and interpersonal climate remain important, but their retention value may be strongest when they reinforce professional meaning, engagement, and value congruence.

This interpretation is supported by recent evidence from Slovakia showing that job satisfaction predicts nurses’ intention to stay with their employer partly through work engagement [34].

The nonsignificant role of place‐related mobility preferences also deserves attention. In the present model, willingness to work abroad, preference for working close to home, and willingness to relocate for a better job offer did not independently explain intention to stay in healthcare. This does not mean that mobility or migration is unimportant for healthcare labor markets. Rather, it suggests that place‐related preferences alone may not be sufficient to explain whether respondents want to remain in the profession or sector. Migration‐related research similarly indicates that mobility intentions among nursing students and nurses are shaped by financial incentives, career opportunities, professional identity, and learning environments rather than by geographic flexibility alone [22–24]. The high similarity between the Czech and Slovak samples is also noteworthy. Although the omnibus country comparison was statistically significant, individual differences were trivial or very small, and the regression pattern was highly comparable. This supports the interpretation that the studied mechanisms may operate similarly in two historically and institutionally proximate healthcare contexts, while still leaving room for future research to test whether the pattern holds in more diverse systems.

Several limitations should be acknowledged. First, the cross‐sectional design does not allow causal inference, and the observed associations should therefore be interpreted as explanatory and correlational rather than causal. Second, intention to stay was measured using a single item. Although Q41 is theoretically relevant as an early indicator of workforce stability, it does not provide the same measurement precision as an established multi‐item intention‐to‐stay or turnover‐intention scale. In addition, the wording of Q41 was conditional, referring to willingness to remain in healthcare if respondents’ preferences are met. The outcome should therefore be interpreted as a conditionally worded indicator of intention to stay rather than as a direct measure of unconditional long‐term retention intention. This limits direct comparability with studies using validated multi‐item retention or turnover‐intention instruments. Third, the sample included respondents connected to both healthcare education and practice. This broader composition is relevant to the study aim, because retention‐related attitudes may begin to form before full professional entry, but it also means that the findings should not be generalized uncritically to fully employed healthcare professionals only. Fourth, the questionnaire was author‐developed, and no separate attention‐check items were embedded. Although the psychometric analyses support exploratory use of the retained scales, future studies should validate the instrument further, include more detailed occupational and employment characteristics, and consider established multi‐item outcome measures. Fifth, mobility was measured as a relatively narrow place‐related construct, which may partly explain its limited effect and suggests the need for more differentiated measures of career mobility, migration aspirations, and organizational embeddedness in future research. Future studies could combine longitudinal designs, more precise mobility indicators, and cross‐national invariance testing to clarify whether the hierarchy observed here remains stable across time and across more diverse health systems [33, 37–41]. Overall, the findings support a cautious conclusion that professional commitment was the strongest predictor within the tested model, while workplace relationships and leadership functioned as an important contextual factor and place‐related mobility preferences did not independently predict intention to stay.

## 5. Conclusion

The present study adds to current retention research by showing that, within the tested cross‐sectional model, professional commitment had the largest association with intention to stay in healthcare, while workplace relationships and leadership had a significant but smaller association, and place‐related mobility preferences showed no independent explanatory role. The comparable pattern observed in the Czech and Slovak samples suggests that these mechanisms may be similar across two closely related Central European healthcare contexts. At the same time, the findings should be interpreted cautiously because the model explained approximately one‐quarter of the variance, the outcome was assessed using a single conditionally worded item, and the sample included respondents connected to both healthcare education and practice. The practical implication is that retention strategies should combine improvements in work and learning environments with systematic support for professional meaning, commitment, recognition, mentoring, and identification with the healthcare role. Long‐term workforce stability in healthcare is therefore likely to depend not only on structural conditions but also on whether current and future healthcare personnel experience their profession as meaningful, valued, and worth continuing [43].

## Funding

The authors received no specific funding for this work

## Ethics Statement

The study was approved by the Ethics Committee of the Faculty of Health Studies, University J. E. Purkyně (No. 2025/12/1_001). This study was conducted in accordance with the Helsinki Declaration and in compliance with publication ethics principles.

## Consent

Participants were provided with both verbal and written information about the study, and written informed consent was obtained prior to participation. Participation was voluntary, and participants were informed that they could withdraw at any time without any consequences.

## Conflicts of Interest

The authors declare no conflicts of interest.

## Supporting Information

Additional supporting information can be found online in the Supporting Information section.

## Supporting information


**Supporting Information** Supporting Information 1: Questionnaire items. Supporting Information 2: Standardized CFA factor loadings.

## Data Availability

The data that support the findings of this study are available from the corresponding author upon reasonable request.
