# Supplementary material for: Predictors of Intention to Stay in Healthcare Among Healthcare Students and Professionals in Czechia and Slovakia
Source: J Nurs Manag. 2026 Aug 7;2026:1300076. doi: 10.1155/jonm/1300076 (PMC13451636; doi:10.1155/jonm/1300076)
Supplement: Supplementary file 1 [file JONM-2026-1300076-s001.docx]

**Supplementary Material 1: Questionnaire items**

# INSTRUCTIONS FOR COMPLETING THE QUESTIONNAIRE

Most questions are answered by circling the appropriate response. Questions No. 2, No. 3, No. 4 are open-ended, i.e. please write in the requested answer. The questionnaire uses a seven-point scale, ranging from “completely disagree” to “completely agree”. The wider range of response options allows you to express the degree of your agreement or disagreement more precisely. We would appreciate it if your answers were truthful and based on your own experience or views.

**Example responses:**

**Gender**

Male

Female

Other

Prefer not to answer

**Year of study**

**2**

**Age**

**22**

**I have work experience in healthcare** (write the number of years or months: indicate Y for years or M for months, e.g. 1M or 2Y)

**3Y**

**The decision to remain in the healthcare sector is a priority for me.**

| Completely disagree | Strongly disagree | Rather disagree | Neither agree nor disagree | Rather agree | Strongly agree | Completely agree |
| --- | --- | --- | --- | --- | --- | --- |
| 1 | 2 | 3 | 4 | 5 | 6 | 7 |

**THE QUESTIONNAIRE BEGINS ON THE NEXT PAGE**

1. **Gender**
   i. Male
   ii. Female
   iii. Other
   iv. Prefer not to answer
2. **Year of study**
3. **Age**
4. **I have work experience in healthcare – excluding faculty placements** (write the number of years or months: indicate Y for years or M for months, e.g. 1M or 2Y)
5. **The decision to remain in the healthcare sector is a priority for me.**

| Completely disagree | Strongly disagree | Rather disagree | Neither agree nor disagree | Rather agree | Strongly agree | Completely agree |
| --- | --- | --- | --- | --- | --- | --- |
| 1 | 2 | 3 | 4 | 5 | 6 | 7 |

6. **Family tradition plays an important role in my decision to work in healthcare.**

| Completely disagree | Strongly disagree | Rather disagree | Neither agree nor disagree | Rather agree | Strongly agree | Completely agree |
| --- | --- | --- | --- | --- | --- | --- |
| 1 | 2 | 3 | 4 | 5 | 6 | 7 |

7. **I see my career as being more likely abroad than in my home country.**

| Completely disagree | Strongly disagree | Rather disagree | Neither agree nor disagree | Rather agree | Strongly agree | Completely agree |
| --- | --- | --- | --- | --- | --- | --- |
| 1 | 2 | 3 | 4 | 5 | 6 | 7 |

8. **I perceive work in healthcare as meaningful.**

| Completely disagree | Strongly disagree | Rather disagree | Neither agree nor disagree | Rather agree | Strongly agree | Completely agree |
| --- | --- | --- | --- | --- | --- | --- |
| 1 | 2 | 3 | 4 | 5 | 6 | 7 |

9. **Helping people is an important part of work for me.**

| Completely disagree | Strongly disagree | Rather disagree | Neither agree nor disagree | Rather agree | Strongly agree | Completely agree |
| --- | --- | --- | --- | --- | --- | --- |
| 1 | 2 | 3 | 4 | 5 | 6 | 7 |

10. **The level of financial remuneration motivates me to work in healthcare.**

| Completely disagree | Strongly disagree | Rather disagree | Neither agree nor disagree | Rather agree | Strongly agree | Completely agree |
| --- | --- | --- | --- | --- | --- | --- |
| 1 | 2 | 3 | 4 | 5 | 6 | 7 |

11. **The opportunity for further education and career development motivates me to remain in healthcare.**

| Completely disagree | Strongly disagree | Rather disagree | Neither agree nor disagree | Rather agree | Strongly agree | Completely agree |
| --- | --- | --- | --- | --- | --- | --- |
| 1 | 2 | 3 | 4 | 5 | 6 | 7 |

12. **I perceive clearly defined work goals in healthcare.**

| Completely disagree | Strongly disagree | Rather disagree | Neither agree nor disagree | Rather agree | Strongly agree | Completely agree |
| --- | --- | --- | --- | --- | --- | --- |
| 1 | 2 | 3 | 4 | 5 | 6 | 7 |

13. **I prefer to work independently, without the need to cooperate with others.**

| Completely disagree | Strongly disagree | Rather disagree | Neither agree nor disagree | Rather agree | Strongly agree | Completely agree |
| --- | --- | --- | --- | --- | --- | --- |
| 1 | 2 | 3 | 4 | 5 | 6 | 7 |

14. **I consider work in healthcare to be stable.**

| Completely disagree | Strongly disagree | Rather disagree | Neither agree nor disagree | Rather agree | Strongly agree | Completely agree |
| --- | --- | --- | --- | --- | --- | --- |
| 1 | 2 | 3 | 4 | 5 | 6 | 7 |

15. **The opportunity to participate in decision-making increases my motivation to work.**

| Completely disagree | Strongly disagree | Rather disagree | Neither agree nor disagree | Rather agree | Strongly agree | Completely agree |
| --- | --- | --- | --- | --- | --- | --- |
| 1 | 2 | 3 | 4 | 5 | 6 | 7 |

16. **Benefits, such as a scholarship or housing support, influence my decision to take up employment.**

| Completely disagree | Strongly disagree | Rather disagree | Neither agree nor disagree | Rather agree | Strongly agree | Completely agree |
| --- | --- | --- | --- | --- | --- | --- |
| 1 | 2 | 3 | 4 | 5 | 6 | 7 |

17. **I do not consider helping people to be a key reason why I work in healthcare.**

| Completely disagree | Strongly disagree | Rather disagree | Neither agree nor disagree | Rather agree | Strongly agree | Completely agree |
| --- | --- | --- | --- | --- | --- | --- |
| 1 | 2 | 3 | 4 | 5 | 6 | 7 |

18. **I prefer a part-time position so that I have more free time.**

| Completely disagree | Strongly disagree | Rather disagree | Neither agree nor disagree | Rather agree | Strongly agree | Completely agree |
| --- | --- | --- | --- | --- | --- | --- |
| 1 | 2 | 3 | 4 | 5 | 6 | 7 |

19. **I prefer cooperation to an authoritative approach from supervisors.**

| Completely disagree | Strongly disagree | Rather disagree | Neither agree nor disagree | Rather agree | Strongly agree | Completely agree |
| --- | --- | --- | --- | --- | --- | --- |
| 1 | 2 | 3 | 4 | 5 | 6 | 7 |

20. **Cooperation and shared responsibility with colleagues motivate me.**

| Completely disagree | Strongly disagree | Rather disagree | Neither agree nor disagree | Rather agree | Strongly agree | Completely agree |
| --- | --- | --- | --- | --- | --- | --- |
| 1 | 2 | 3 | 4 | 5 | 6 | 7 |

21. **Working in a positive team is important to me.**

| Completely disagree | Strongly disagree | Rather disagree | Neither agree nor disagree | Rather agree | Strongly agree | Completely agree |
| --- | --- | --- | --- | --- | --- | --- |
| 1 | 2 | 3 | 4 | 5 | 6 | 7 |

22. **I care about work-life balance.**

| Completely disagree | Strongly disagree | Rather disagree | Neither agree nor disagree | Rather agree | Strongly agree | Completely agree |
| --- | --- | --- | --- | --- | --- | --- |
| 1 | 2 | 3 | 4 | 5 | 6 | 7 |

23. **Trust between supervisors and colleagues is essential to me.**

| Completely disagree | Strongly disagree | Rather disagree | Neither agree nor disagree | Rather agree | Strongly agree | Completely agree |
| --- | --- | --- | --- | --- | --- | --- |
| 1 | 2 | 3 | 4 | 5 | 6 | 7 |

24. **It is important to me that the organizational culture corresponds to my values.**

| Completely disagree | Strongly disagree | Rather disagree | Neither agree nor disagree | Rather agree | Strongly agree | Completely agree |
| --- | --- | --- | --- | --- | --- | --- |
| 1 | 2 | 3 | 4 | 5 | 6 | 7 |

25. **Recognition for work well done is important to me.**

| Completely disagree | Strongly disagree | Rather disagree | Neither agree nor disagree | Rather agree | Strongly agree | Completely agree |
| --- | --- | --- | --- | --- | --- | --- |
| 1 | 2 | 3 | 4 | 5 | 6 | 7 |

26. **I appreciate it when supervisors act as mentors.**

| Completely disagree | Strongly disagree | Rather disagree | Neither agree nor disagree | Rather agree | Strongly agree | Completely agree |
| --- | --- | --- | --- | --- | --- | --- |
| 1 | 2 | 3 | 4 | 5 | 6 | 7 |

27. **I expect supervisors to listen to and respect my opinions.**

| Completely disagree | Strongly disagree | Rather disagree | Neither agree nor disagree | Rather agree | Strongly agree | Completely agree |
| --- | --- | --- | --- | --- | --- | --- |
| 1 | 2 | 3 | 4 | 5 | 6 | 7 |

28. **Work should always take priority over my personal life.**

| Completely disagree | Strongly disagree | Rather disagree | Neither agree nor disagree | Rather agree | Strongly agree | Completely agree |
| --- | --- | --- | --- | --- | --- | --- |
| 1 | 2 | 3 | 4 | 5 | 6 | 7 |

29. **I value open and transparent communication in the workplace.**

| Completely disagree | Strongly disagree | Rather disagree | Neither agree nor disagree | Rather agree | Strongly agree | Completely agree |
| --- | --- | --- | --- | --- | --- | --- |
| 1 | 2 | 3 | 4 | 5 | 6 | 7 |

30. **I appreciate regular interest and contact from supervisors.**

| Completely disagree | Strongly disagree | Rather disagree | Neither agree nor disagree | Rather agree | Strongly agree | Completely agree |
| --- | --- | --- | --- | --- | --- | --- |
| 1 | 2 | 3 | 4 | 5 | 6 | 7 |

31. **I prefer employment in the public healthcare sector.**

| Completely disagree | Strongly disagree | Rather disagree | Neither agree nor disagree | Rather agree | Strongly agree | Completely agree |
| --- | --- | --- | --- | --- | --- | --- |
| 1 | 2 | 3 | 4 | 5 | 6 | 7 |

32. **I want to work close to where I live.**

| Completely disagree | Strongly disagree | Rather disagree | Neither agree nor disagree | Rather agree | Strongly agree | Completely agree |
| --- | --- | --- | --- | --- | --- | --- |
| 1 | 2 | 3 | 4 | 5 | 6 | 7 |

33. **I am willing to relocate if I receive a better job offer.**

| Completely disagree | Strongly disagree | Rather disagree | Neither agree nor disagree | Rather agree | Strongly agree | Completely agree |
| --- | --- | --- | --- | --- | --- | --- |
| 1 | 2 | 3 | 4 | 5 | 6 | 7 |

34. **I value regular and structured feedback on my work.**

| Completely disagree | Strongly disagree | Rather disagree | Neither agree nor disagree | Rather agree | Strongly agree | Completely agree |
| --- | --- | --- | --- | --- | --- | --- |
| 1 | 2 | 3 | 4 | 5 | 6 | 7 |

35. **It does not matter to me whether I am regularly rewarded for my work.**

| Completely disagree | Strongly disagree | Rather disagree | Neither agree nor disagree | Rather agree | Strongly agree | Completely agree |
| --- | --- | --- | --- | --- | --- | --- |
| 1 | 2 | 3 | 4 | 5 | 6 | 7 |

36. **I prefer trust and independence in work tasks.**

| Completely disagree | Strongly disagree | Rather disagree | Neither agree nor disagree | Rather agree | Strongly agree | Completely agree |
| --- | --- | --- | --- | --- | --- | --- |
| 1 | 2 | 3 | 4 | 5 | 6 | 7 |

37. **I consider equal conditions for all employees important.**

| Completely disagree | Strongly disagree | Rather disagree | Neither agree nor disagree | Rather agree | Strongly agree | Completely agree |
| --- | --- | --- | --- | --- | --- | --- |
| 1 | 2 | 3 | 4 | 5 | 6 | 7 |

38. **When making decisions at work, I rely on the opinions of more experienced colleagues.**

| Completely disagree | Strongly disagree | Rather disagree | Neither agree nor disagree | Rather agree | Strongly agree | Completely agree |
| --- | --- | --- | --- | --- | --- | --- |
| 1 | 2 | 3 | 4 | 5 | 6 | 7 |

39. **I expect regular remuneration for my work.**

| Completely disagree | Strongly disagree | Rather disagree | Neither agree nor disagree | Rather agree | Strongly agree | Completely agree |
| --- | --- | --- | --- | --- | --- | --- |
| 1 | 2 | 3 | 4 | 5 | 6 | 7 |

40. **I prefer fewer internal rules and less administration.**

| Completely disagree | Strongly disagree | Rather disagree | Neither agree nor disagree | Rather agree | Strongly agree | Completely agree |
| --- | --- | --- | --- | --- | --- | --- |
| 1 | 2 | 3 | 4 | 5 | 6 | 7 |

41. **If my preferences are met, I want to remain in healthcare.**

| Completely disagree | Strongly disagree | Rather disagree | Neither agree nor disagree | Rather agree | Strongly agree | Completely agree |
| --- | --- | --- | --- | --- | --- | --- |
| 1 | 2 | 3 | 4 | 5 | 6 | 7 |

**Supplementary Material 2: Standardized CFA factor loadings**

Table S1 presents standardized factor loadings from the three-factor confirmatory factor analysis model. The table is included to facilitate interpretation of the CFA results. Q41 was retained in the measurement model because it loaded on the professional-commitment factor, but it was used exclusively as the dependent variable and was excluded from the professional-commitment predictor composite in the regression analyses.

Table S1. Standardized factor loadings from the confirmatory factor analysis

| Latent factor | Item | Item wording | Standardized loading |
| --- | --- | --- | --- |
| Workplace relationships and leadership | Q20 | Cooperation and shared responsibility with colleagues motivate me. | 0.504 |
| Workplace relationships and leadership | Q21 | Working in a positive team is important to me. | 0.753 |
| Workplace relationships and leadership | Q22 | I care about work-life balance. | 0.549 |
| Workplace relationships and leadership | Q23 | Trust between supervisors and colleagues is essential to me. | 0.760 |
| Workplace relationships and leadership | Q29 | I value open and transparent communication in the workplace. | 0.622 |
| Workplace relationships and leadership | Q30 | I appreciate regular interest and contact from supervisors. | 0.593 |
| Professional commitment | Q5 | The decision to remain in the healthcare sector is a priority for me. | 0.701 |
| Professional commitment | Q8 | I perceive work in healthcare as meaningful. | 0.605 |
| Professional commitment | Q11 | The opportunity for further education and career development motivates me to remain in healthcare. | 0.557 |
| Professional commitment | Q12 | I perceive clearly defined work goals in healthcare. | 0.567 |
| Professional commitment | Q41 | If my preferences are met, I want to remain in healthcare. | 0.661 |
| Mobility | Q7 | I see my career as being more likely abroad than in my home country. | 0.557 |
| Mobility | Q32 | I want to work close to where I live. | 0.530 |
| Mobility | Q33 | I am willing to relocate if I receive a better job offer. | 0.808 |

Note. Loadings are shown in standardized form. Q32 is reported under the mobility factor in accordance with the measurement model. In the regression analyses, Q41 was excluded from the professional-commitment predictor composite to avoid criterion contamination. All standardized loadings were of moderate to high magnitude, supporting substantive interpretation of the three-factor structure, although the global CFA fit was interpreted as borderline rather than optimal in the manuscript.
